# The role of antenatal relaxation practices in enhancing maternal psychological wellbeing and childbirth experiences: an observational study

**DOI:** 10.3389/fgwh.2025.1597174

**Published:** 2025-05-16

**Authors:** Mo Tabib, Tracy Humphrey, Katrina Forbes-McKay

**Affiliations:** ^1^ School of Health, Robert Gordon University, Aberdeen, United Kingdom; ^2^ School of Health and Social Care, Edinburgh Napier University, Sighthill Campus, Edinburgh, United Kingdom; ^3^ Clinical and Health Sciences, University of South Australia, City East Campus, Adelaide, SA, Australia

**Keywords:** antenatal education, relaxation practices, perinatal psychological wellbeing, childbirth self-efficacy, fear of childbirth, anxiety, childbirth experiences

## Abstract

**Introduction:**

There is growing qualitative evidence that antenatal education on relaxation practices can enable women to deliberately induce a deep state of emotional calmness. Learning to shift focus from distressing emotions such as anxiety and fear to this altered state of calmness may significantly enhance women's confidence, thereby protecting maternal psychological wellbeing and leading to more positive childbirth experiences. However, the generalisability of these findings remains uncertain. This study aimed to bridge this gap by using quantitative methods to validate and extend the qualitative evidence.

**Methods:**

Through an observational study with a prospective longitudinal cohort design, ninety-one women attending a single antenatal relaxation class at a Scottish NHS maternity service completed online surveys including Childbirth Self-Efficacy Inventory (CBSEI), Warwick Edinburgh Mental Well-Being Scale (WEMWBS), Wijma Delivery Expectancy/Experience Questionnaire (W-DEQ), and Six-item State-Trait Anxiety Inventory (STAI-6) at pre-class, post-class and post-birth.

**Results:**

Findings indicated significant improvements in childbirth self-efficacy expectancy, mental wellbeing, fear of childbirth, and both trait and state anxiety after attending the class, and these improvements remained stable until 4–8 weeks after birth. Women widely reported using relaxation practices, with the majority perceiving a positive influence on their pregnancy and childbirth experiences. The majority also viewed their overall childbirth experiences as positive.

**Discussion:**

Consequently, maternity services should consider reforming current antenatal education to align with these findings.

## Introduction

Antenatal education on relaxation practices intends to empower women to deliberately induce a deep state of emotional calmness. Qualitative research suggests that women who engage in these relaxation practices can shift their focus from distressing emotions such as fear and anxiety to an altered state of calmness (1–4). This shift seems to enhance their confidence towards childbirth and promote positive childbirth experiences (1–3, 5). In a recent study conducted by Tabib et al. (4) women reported feeling less fearful and anxious, and more confident towards childbirth after attending a three-hour relaxation class. Participants also described experiencing a sense of wellness and positivity, which was maintained during pregnancy and childbirth. However, the generalisability of these qualitative findings to larger populations remains to be fully explored.

Although quantitative research studies demonstrate that antenatal education on relaxation practices can effectively reduce fear of childbirth (FOC) (6–9) and antenatal anxiety (10–15), the influence on postnatal anxiety remains under-explored. In addition, despite the existing evidence on the effect of relaxation practices on reducing childbirth related fear and anxiety, a gap in evidence remains regarding their impact on childbirth self-efficacy and maternal mental wellbeing, especially in the context of Western countries. This is largely due to the prevailing pathogenesis paradigm in medical and health sciences, which focuses mainly on identifying the origins of disease and risk factors (16). In contrast, the salutogenesis paradigm, introduced by Antonovsky in 1979, emphasises the origins of health and the assets that promote it. This theory of health promotion shifts the focus from treating disease to the enhancement of overall wellbeing. It considers not only negative emotions like fear and anxiety but also positive psychological constructs such as self-efficacy and mental wellbeing, which are indicators of general wellbeing.

Therefore, this study addresses the identified knowledge gap by adopting a salutogenic approach. It seeks to examine the influence of attending an antenatal education class incorporating relaxation practices, on self-efficacy, mental wellbeing, fear of childbirth, and anxiety over time. In addition, the study aims to evaluate women's perceptions of how receiving education on relaxation practices may influence their childbirth experiences.

### Aims

This study examines the influence of a single online Antenatal Relaxation Class (ARC), an established initiative in a Scottish NHS Health Board, on maternal psychological wellbeing and childbirth experiences. It aims to assess changes in childbirth self-efficacy, mental wellbeing, fear of childbirth, and state and trait anxiety over time (pre-class, post-class and post-birth). Further, it explores women's perspectives on the influence of ARC on their pregnancy, labour and birth experiences as well as their perceptions of their overall childbirth experiences.

## Method

### Design

The study employed a prospective longitudinal cohort design, using online surveys for data collection at pre-class, post-class and post-birth.

### Setting

The study setting was a specialist maternity hospital in an NHS Health Board in North-East of Scotland with around 5,000 births per annum. The setting was selected due to its existing provision of this single session of ARC.

### Antenatal relaxation class (ARC)

Antenatal educational interventions on relaxation practices, as utilised in previous studies (6, 9, 17, 18) are often too lengthy and costly for implementation in under-pressure national health services. In contrast, some studies with low-cost and brief interventions (11, 19, 20), involved multiple sessions though some participants did not attend all subsequent session. Therefore, a single, low-cost session, as opposed to costly or multiple sessions can facilitate the intended delivery of the education, reduce attrition rates, and make it more affordable for national health services such as the UK NHS maternity services.

ARC is an established initiative within a Scottish NHS Health Board. This single, 3-hour class was delivered online to groups of 4–12 women and facilitated by two midwives, independent of the research team, who were trained in relaxation techniques. ARC was offered to all pregnant women and their birth partners in the third trimester. However, attendance of women expressing anxiety or apprehension of childbirth was actively encouraged by their maternity care providers. Women attended the class from their home. In class, after an introduction to the effect of emotions on childbirth physiology, women practised four relaxation exercises including breathing, visualisation, hypnosis, and relaxation in labour. Women were also given leaflets and audio resources for further practice at home. The class and provided resources were free of charge for women and their partners. Further details about ARC can be found in Tabib et al. (4).

### Participants and sampling

Convenience sampling was used to invite all women on the waiting list for an antenatal relaxation class between January and June 2021 (243 women) to participate in the study. The inclusion criteria included being aged 16 or over, and being able to read, write and understand English. Women were excluded if they had significant mental health issue requiring medication or did not meet the inclusion criteria. Based on a G*power calculation, a sample of 57 participants was required to detect treatment effects, assuming power = 0.95, significance set to 0.05, and an effect size of d = 0.4.

### Measurements

The Childbirth Self-Efficacy Inventory (CBSEI) (21) was selected to measure self-efficacy expectancy and outcome expectancy in labour based on Bandura's self-efficacy theory (22). The CBSEI is a reliable and valid instrument with Cronbach's alpha scores of 0.90 (23) which has been used in multiple studies of pregnant women. Efficacy expectancy is a personal conviction about one's ability to successfully perform required behaviours in a given situation, and outcome expectancy is the belief that a given behaviour will lead to a given outcome (24). In this study, a 30-item version of CBSEI was used with the same 15 items measuring efficacy expectance and outcome expectancy. Higher scores indicate a higher degree of CBSE with maximum scores being set to 150.

The Warwick Edinburgh Mental Well-Being Scale (WEMWBS) (25, 26), a psychometrically validated tool, was included to measure subjective well-being (e.g., “I have been feeling useful”) and psychological functioning (e.g., “I've been dealing with problems”). WEMWBS demonstrates high content validity with Cronbach's alpha scores of around 0.90 in studies of the general population and pregnant women (25). The minimum scale score is 14 and the maximum is 70, with a higher score indicating a higher level of mental wellbeing.

The Wijma Delivery Expectancy/Experience Questionnaire (W-DEQ) (27), version A (W-DEQ A) and version B (W-DEQ B) were used to measure fear of childbirth. W-DEQ A was administered before and after class to measure fear related to the upcoming childbirth during pregnancy and W-DEQ B was used post-birth to evaluate fear of childbirth after birth (27). Both versions A and B include 33 items with items ranging from 0 (extremely) to 5 (not at all). This instrument is a well-validated tool with Cronbach's alpha of 0.92 (23). Korukcu et al. (28) established CBSEI cut-off scores as follows: a score of 0–60 indicates low fear, 61–84 indicates moderate fear, and a score of 85 or above indicates severe fear of childbirth.

The Six-item State-Trait Anxiety Inventory (STAI-6) (29) was used to measure participants’ emotional reactions, assessing anxiety at a specific moment (state anxiety) and the general response to perceived threats (trait anxiety). The STAI is a reliable and valid self-report measure that has obtained scores similar to the full STAI in pregnant women (29). Statements are scored on a 4-point scale of increasing intensity, from “*not at all*” to “*very much so*” (with scores of 1–4 respectively).

Although a threshold point for high anxiety has not been properly deﬁned, most studies consider a score above 12 in 6-item STAI as being highly anxious (30).

The study-specific questionnaire included questions regarding age, gestational age or postnatal days at the time of survey completion, parity, ethnicity, marital status, educational attainment and employment status. It also included 5-point Likert scales ranging from “very negatively” to “very positively” to measure the participants’ perspectives on the influence of attending ARC on their pregnancy and labour/birth experiences, and to gauge their perceptions on the quality of their overall childbirth experiences. Table 1 presents the selection of the measurement tools and the time points for data collection.

**Table 1:** Selection of measurement tools.

Measure	Timing
CBSEI (childbirth self-efficacy including outcome expectancy & self-efficacy expectancy)	Pre-class and post- class
WEMWBS (mental wellbeing)	Pre-class, post-class, and post-birth
STAI-6 (state & trait anxiety)	
W-DEQ (fear of Childbirth)	
Likert scale questions on the influence of ARC on experiences of pregnancy, labour, and birth	Post-class and post-birth
Likert scale question on the overall experience of childbirth	Post-birth

The table presents the selection of the measurement tools and the time points for data collection.

### Data collection

The data were collected via Novi Online survey. A link to the participant information sheet, consent form and survey was emailed to all women on the waiting list by the midwives facilitating ARC.

### Data analysis

The data were analysed using Statistics Package for the Social Sciences (SPSS), Version 25 (31). Descriptive statistics including frequency count, percentage, mean and standard deviation, were conducted to ascertain sample characteristics and address the aims of the study. One-way repeated measures analysis of variance (ANOVA) was used to explore the impact of time (pre-class, post-class, and post-birth) on FOC, state and trait anxiety and mental wellbeing. Bonferroni adjustment to the alpha level 0.05 was made for the comparisons taken place via one-way measured ANOVA. *post-hoc* comparisons were used to explore the differences between the measures at different time points. Since CBSE was only measured at two time points, a paired samples *t*-test was carried out to analyse the difference between CBSE at pre-class and post-class. The authors used a 95% confidence level, and a *p*-value of 0.05 or less was deemed to be significant.

### Ethics statement

The participants provided informed written consent, and full ethical approval was granted by the National Research Ethics Service (REC reference number: 17/LO/0666).

Participation in the study was voluntary, and participants could withdraw from the study at any point prior to the completion of data collection, without giving any reason. Participants were assured that their responses would remain confidential unless there was a disclosure of intent to harm themselves or others. There were no breaches of confidentiality.

## Results

### Sample characteristics

Out of 243 women invited to participate in the study, 91 completed the pre-class survey, resulting in a response rate of 37%. Around two weeks after the class, 85 women (93.4%) returned the post-class survey, and 84 women (92.4%) completed the post-birth survey 4–8 weeks after giving birth. This led to an attrition rate of 6.6% for the post-class survey and 7.6% for the post-birth survey.

Participants’ age ranged from 21 to 41 years (*M* = 31.00, *SD* = 3.6). Mean gestation was 31.7 weeks (*SD* = 3.2) at pre-class, 34.00 weeks (*SD* = 3.3) at post-class and 5.00 weeks postnatal (*SD* *=* *2.4*) at post-birth. The sample characteristics of the participants are presented in Table 2. The participants were predominantly primigravida (*n* = 78, 85.7%) and from a range of ethnicities with the majority (*n* = 70, 76.9%) being white British. A large proportion of the participants were either married (*n* = 56, 61.5%) or co-habiting (*n* = 29, 32.9%) with the remaining (*n* = 6, 6.6%) identifying themselves as single. In terms of educational attainment, this varied from secondary school to doctorate with 79.1% (*n* = 72) being educated to first level degree of higher education or higher. Most participants (*n* = 76, 83.3%) were in full-time employment, whilst 7.7% (*n* = 7) were in part-time employment and the rest were unemployed, students, or others.

**Table 2:** Demographic characteristics.

Characteristic	*n* (%)
Previous births	
No previous birth	78 (85.7%)
One previous birth	11 (12.1%)
Two or more previous birth	2 (2.2%)
Ethnicity	
White British	70 (76.9%)
Other White	10 (10%)
Black	0 (0%)
Asian	7 (7.7%)
Mixed	3 (3.3%)
Others	1 (1.1%)
Marital status	
Married	56 (61.5%)
Domestic partnership	29 (31.9%)
Single	6 (6.6%)
Educational attainment	
Secondary school	4 (4.4%)
College (HNS/HND)	15 (16.5%)
Degree	39 (42.9%)
Master's degree	25 (27.5%)
Doctorate	3 (3.3%)
Others	5 (5.5%)
Employment	
Full-time	76 (83.5%)
Part-time	7 (7.7%)
Unemployed	5 (5.5%)
Student	1 (1.1%)
Others	2 (2.2%)

The table displays demographic characteristics of the study participants, including parity, ethnicity, marital status, educational attainment and employment.

### Childbirth self-efficacy

Results from a paired-sample t-test indicated that participants reported significantly higher mean scores of self-efficacy expectancy at post-class than those reported pre-class *t* (77) = 9.44, *p* < .001, Hedges’ g = 1.6 (indicating a large effect size). However, no statically significant difference was found between the mean scores of outcome-expectancy at pre-class and post-class *t* (78) = −1.51, *p* = 0.135, Hedges’ g = 0.17 (indicating a small effect size). Therefore, results indicate although women's beliefs that their coping strategies would lead to positive outcomes did not significantly change, their confidence in their ability to remain in control during labour increased after the class.

### Mental wellbeing

As shown in Table 3, results from one-way ANOVA indicate that the mean score for mental wellbeing significantly increased at post-class and post-birth compared with pre-class with a large effect size [Wilks Lambda = 0.73, F (2, 72) = 12.32, *p* < 0.001, multivariate partial Eta squared = 0.26]. *post hoc* analysis indicated significant increases between pre-class and post-class (*p* = .000), and between pre-class and post-birth (*p* = .01).

**Table 3:** Mean (SD) for efficacy-expectancy, outcome-expectancy, mental wellbeing, FOC, and state and trait anxiety over time.

Measured parameter	Pre-class (*n* = 91) mean (*SD*)	Post-class (*n* = 85) mean (*SD*)	Post-birth (*n* = 84) mean (*SD*)	*p* Value between pre-class and post-class	*p* Value between pre-class and post-birth
Efficacy-expectancy	84.63 (28.29)	110.56 (23.36)	————	<.001	————
Outcome-expectancy	127.00 (19.30)	129.58 (19.66)	————	=0.135	————
Mental wellbeing	50.36 (7.96)	53.43 (6.58)	52.86 (6.81)	=.000	=.01
Fear of childbirth	62.19 (20.87)	47.89 (21.04)	46.92 (27.19)	<0.001	<0.001
State anxiety	12.43 (3.79)	10.43 (3.37)	9.70 (3.37)	<0.001	<0.001
Trait anxiety	13.06 (3.54)	11.49 (3.31)	11.59 (3.42)	< 0.001	=0.002

The table presents mean (*SD*) for efficacy-expectancy, outcome-expectancy**,** mental wellbeing, FOC, and state and trait anxiety over time.

### Fear of childbirth

As shown in Table 3, results from one-way ANOVA indicate that the mean score for fear of childbirth decreased at post-class and post-birth compared with pre-class with a large effect size [Wilks Lambda = 0.55, F (2, 72) = 27.79, *p* < 0.001, multivariate partial Eta squared = 0.44]. *post hoc* analysis indicates significant decreases in fear of childbirth between pre-class and post-class (*p* < 0.001) and between pre-class and post-birth (*p* < 0.001).

### State anxiety

One-way ANOVA results show that the mean score for state anxiety decreased at post-class and post-birth compared with pre-class with a large effect size [Wilks Lambda = 0.60, F (2, 72) = 22.17, *p* < 0.001, multivariate partial Eta squared = 0.39]. *post hoc* analysis indicated significant decreases in state anxiety between pre-class and post-class (*p* < 0.001) and between pre-class and post-birth (*p* < 0.001).

### Trait anxiety

One-way ANOVA results show that the mean score for trait anxiety decreased at post-class and post-birth compared with pre-class with a large effect size [Wilks Lambda = 0.70, F (2, 72) = 14.24, *p* < 0.001, multivariate partial Eta squared = 0.29]. *post hoc* analysis indicated significant decreases in trait anxiety between pre-class and post-class (*p* < 0.001) and between pre-class and post-birth (*p* = 0.002).

The results indicate that there is a significant effect of time on childbirth self-efficacy expectancy, mental wellbeing, fear of childbirth, and state and trait anxiety. Significant improvements in these parameters were observed post-class and maintained until post-birth. Table 3 presents changes in childbirth self-efficacy, mental wellbeing, fear of childbirth and anxiety over time.

### Influence of ARC on pregnancy, labour and birth experiences

The majority of participants reported wide practice of relaxation techniques in pregnancy (95.2%, *n* = 80) and during labour and/or birth (94.0%, *n* = 79). Most participants (97.6%, *n* = 82) perceived the influence of ARC on their experience of pregnancy as either “positive” (63.1%, n = 53) or “very positive” (34.5%, *n* = 29). All women (100%) who had experienced labour (89.3%, *n* = 75) reported using the techniques in labour. Interestingly, some of those who underwent elective caesarean section also reported using the techniques during the procedure. Over 80% (*n* = 71) of them perceived the influence of ARC on their labour and birth experience as “positive” (56.0%, *n* = 47) or “very positive” (28.6%, *n* = 24), whilst 15.5% (*n* = 13) felt attending ARC had “no influence” on their labour and birth experience. None of the participants reported ARC as having a “negative” or “very negative” influence on either their pregnancy or labour and birth.

### Overall childbirth experiences

The majority (73.8%, *n* = 62) of those who returned the post-birth survey, perceived their overall labour and birth experience as “positive” or “very positive”, 9.5% (*n* = 8) expressed having overall negative experiences and 16.7% (*n* = 14) perceived their experience as “neither positive nor negative”. None of the participants reported having a “very negative” labour or birth experience.

However, these findings need to be interpreted in view of the disparity between study participants’ expected mode and place of birth (data collected in pregnancy) and their actual mode and place of birth. Results indicated a disparity between the expected (reported post-class) and actual (reported post-birth) mode and place of birth. Despite the majority of women expecting to give birth spontaneously (*n* = 75, 88.2%) in the midwife-led units (*n* = 54, 63.6%) at post-class, only around one-third of these women reported meeting their expectations in terms of mode (*n* = 28, 33.3%) and place (*n* = 23, 27.4%) of birth.

To conclude, whilst around two-thirds of women did not meet their expectations in terms of mode and place of birth, only 9.5% perceived their overall labour and birth experience as “negative” and no one reported having a “very negative” experience.

## Discussion

The study aimed to assess changes in women's childbirth self-efficacy, mental wellbeing, fear of childbirth and anxiety after attending ARC, and to evaluate if women perceived ARC's influence and their overall childbirth experiences positively. The findings indicated that attending ARC was associated with significant improvements in childbirth self-efficacy expectancy, mental wellbeing, fear of childbirth and state and trait anxiety. These improvements remained stable until after the birth. Women reported widely using relaxation practices and viewed the influence on their pregnancy and childbirth experiences as positive. The majority reported having an overall positive childbirth experience, even though around two-thirds did not meet their expectations in terms of mode or place of birth. These findings complement the existent qualitative evidence in the field and meet some of the gaps in the literature.

Whilst qualitative evidence consistently suggests that antenatal education incorporating relaxation practices can boost women's confidence in their birthing abilities (1–4), quantitative research supporting the generalisability of this finding is lacking, particularly in Western countries. The two comparative studies that reported the effect of such education on self-efficacy (6, 9) were conducted in Turkey, and similar to our findings, found a significant increase in efficacy expectancy at post-education compared with baseline. In contrast with these two studies, our findings did not show a significant increase in outcome expectancy. In our study, women were already scoring highly on outcome expectancy at baseline. This meant that they already had strong beliefs that their coping behaviours such as using relaxation practices during labour would produce desired outcomes (outcome-expectancy). At baseline, however, they were not confident that they could perform these behaviours during labour (efficacy-expectancy). Bandura (24) argues that although people may believe that a certain behaviour will enable them to cope in a given situation (outcome-expectancy), this may not influence behaviour if they do not believe they can perform it (efficacy-expectancy). This has significant implications for antenatal education, rather than educating women about the techniques that are useful during labour, women need to build their confidence in using and mastering such techniques.

Finding a significant reduction in mean scores of FOC is congruent with the previous research (6–9). However, all these studies were carried out in Turkey. The only study in the context of the UK was a randomised control trial (RCT) conducted by Downe et al. (11) that compared the expected fear of labour (measured at baseline before attending hypnosis sessions) with the actual levels reported 2 weeks postnatal. The results demonstrated a significant reduction in fear levels. However, a well-validated tool like W-DEQ was not used to measure FOC in the study, which may limit the validity of this finding. Overall, evidence on the influence of education on FOC in the context of Western countries is lacking. Thus, the findings of the present study add new knowledge to this under-investigated area in Western countries.

The reduced levels of antenatal anxiety, following attending ARC, in the present study strengthens existing evidence (10, 12, 15, 32–34). However, a paucity of evidence regarding the effect of such education on postnatal anxiety is evident in the literature and the findings of the current study have made a unique contribution by indicating that improvements seen post-class are maintained post-birth.

This research appears to be the first study to assess the influence of antenatal education incorporating relaxation practices on maternal mental wellbeing, using WEMWBS, both antenatally and postnatally. It is plausible that the prevalent use of relaxation techniques along with highly positive perceptions of their effect, shown in the study, have played a role in the stability of these findings over time.

Previous research in the field appears to be more focused on assessing the influence on negative emotions such as fear and anxiety. In contrast, the current study by reporting the influence of the education on positive emotions of childbirth self-efficacy and mental wellbeing brings new insight to this area of research. The study has adopted a salutogenesis orientation by attending childbirth self-efficacy and mental wellbeing as two positive psychological concepts (35). The inverse relationship between self-efficacy and fear/anxiety is well documented in the literature (21, 23, 36, 37, 46). Understanding this relationship can have significant implications for future research and practice. If childbirth self- efficacy expectancy is such a prominent factor in reducing childbirth fear and anxiety, the focus of future practice and research should indeed be on promoting and examining this positive psychological parameter.

Most participants reported having an overall positive childbirth experience. This aligns with the findings of a recent randomised control study (38) and a systematic review (39), both of which concluded that antenatal hypnosis classes can enhance overall childbirth experience. The percentage of women reporting a negative childbirth experience in the literature varies from 7% to 33.3% (40). Dissatisfaction with childbirth experiences seems to rise when childbirth expectations and outcomes do not match (41–44). As such, the 9.5% of women in this study describing their childbirth experience as negative, appears to be at the lower end of this spectrum, especially given that most did not meet their childbirth expectations.

Evidence suggests that increased childbirth self-efficacy expectancy may have played a role in more satisfaction with childbirth experience (2, 27). Based on the study findings and existing evidence, it is plausible that antenatal education on relaxation practices can enhance women's confidence in their childbirth abilities, improve psychological wellbeing and protect them against experiencing negative emotions like fear and anxiety, leading to more positive childbirth experiences.

## Strengths and limitations

This research makes a unique contribution to existing evidence by providing new evidence in areas that have not previously been explored. It appears to be the first to assess the influence of antenatal relaxation education on perinatal mental wellbeing using WEMWBS, a psychometrically validated tool. Additionally, it seems to be the first study in the UK to examine the effects of such education on fear of childbirth (using W-DEQ) and self-efficacy. The study provides new insight into childbirth experiences, enhancing our understanding of how women's learning from ARC can be materialised in the realities of contemporary maternity services and practices. Furthermore, the high retention rate of study participants has strengthened the internal validity of the study, allowing for robust conclusions. Finally, this study is the first in the field that investigates the influence of a single antenatal relaxation class, which prevents attrition, ensures education is delivered as intended, and makes it more affordable for future research replication.

The study has some limitations. Evidence generated by the study due to its observational design can only establish correlation between attending ARC and the reported changes, and not causality (45). To examine causal relationships, large multicentre and well-designed RCTs are needed to compare the changes over time between the intervention and control groups. Conducting the study in diverse countries would enhance cross-cultural validity. Future research should investigate the longer-term impact of the education on the mental wellbeing of childbearing women and their offspring.

Additionally, a few factors limit the generalisability of the findings. For instance, the results may be subject to volunteer bias, given that participants volunteered to participate in the study. Furthermore, the study was conducted in a single area of Scotland, which may not be representative of the broader population in Scotland or the UK. These factors may restrict the transferability and generalisability of the findings to other areas.

## Conclusion

Attending a single antenatal relaxation class was associated with improved maternal psychological wellbeing and was perceived to positively influence childbirth experiences. Thus, offering such classes to childbearing women/people as a preventative and health-promoting educational programme is recommended.

## Data Availability

The datasets presented in this study can be found in online repositories. The names of the repository/repositories and accession number(s) can be found below: Edinburgh Napier University Research Repository.
